# Data citation needed

**DOI:** 10.1038/s41597-019-0026-5

**Published:** 2019-04-10

## Abstract

Starting last month, publications at *Scientific Data* now include data citations in the main reference list, rather than in a separate data citations section. This change will be supported by changes to the underlying structure of our content to promote machine readability and reuse of links between scholarly articles and datasets. This aligns the journal with a roadmap for data citation co-developed by representatives of the academic community and several publishers, which seeks to make data citation a standard part of the scholarly publishing process.

When the journal was launched in 2014, our separate data citations section was an early and practical implementation of the Joint Declaration of Data Citation Principles^1^, which were signed by many organisations including Nature Publishing Group (the name by which our publisher was previously known). Now that standards have emerged that enable data citations to be robustly included in traditional reference lists, we are pleased to be amongst the first journals to implement this format consistently in our publications. As well as promoting credit for data sharing by enabling citations to datasets to be tracked equivalently to traditional citations, this will also simplify the process of drafting a Data Descriptor, since data citations can now be more easily handled by the same reference management software used for literature references. The first *Scientific Data* paper using this new format was published last month^2^.

The inclusion of data citations in main reference lists is now encouraged by all Nature Research journals as part of their data availability policies. Data citation is also encouraged by nearly 1,600 Springer Nature journals that have adopted standardized research data policy, and by numerous other journals and publishers. All Springer Nature journals, including *Scientific Data*, are also participants in the ‘Initiative for Open Citations’, which means that our reference lists are available as open data making them accessible to the community through services such as CrossRef.

Conceptually and practically, data citation is quite simple. Scholarly reference lists have long included diverse content: books, theses, journal articles, government reports, websites. With online data repositories playing an increasingly common role in research dissemination in all fields, it was well overdue for them to be considered as ‘citeable’ research outputs in their own right (for more see^1,3^). By depositing data in any of *Scientific Data*’s recommended repositories – or any repository that is part of DataCite (https://datacite.org/) – authors will have all the information needed to create a formal data citation.

The data citation roadmap has been published at the journal in three parts. The first piece describes an improved system for creating stable links to datasets at biomedical repositories^4^. The second part lays out a series of data citation guidelines for publishers^5^, which underlie the formatting changes announced here. The third part, published today, releases a series of guidelines for data repositories^6^. These guidelines will help repositories provide consistent advice on how users can cite their records. *Scientific Data* will be progressively incorporating these guidelines into the criteria that we use when selecting repositories to recommend to our authors (http://www.nature.com/sdata/policies/repositories).
